# 

**Published:** 1877-12

**Authors:** 


### CONTENTS:

ARTICLE I.—Efficacy of Electricity in Dental Surgery—Scirrhus and Epithelial Degeneration of the Tongue and Tissues at its Base. By David Prince, M D. .... 337

ARTICLE II.—Outline of a Lecture. By Prof. James B. Hodgkin. D. D. S ..... 343

ARTICLE III—Unity of Force Electricity its Superlative Velocity. By John J. Caldwell, M. D. .... 348

ARTICLE IV.—A few Thoughts Concerning the Treatment of the Lower Bicuspid Teeth. By A. W. Harlan .... 354

ARTICLE V.—The Connection between Excessive Nerve and Brain Worry and Bodily Disease with Pathology and Treatment of Cancers, Tumors, Etc By John J. Caldwell, M. D .... 359

ARTICLE VI.—A Case of Repeated Excision of the Inferior Dental and Gustatory Nerves for the Cure of Dental Neuralgia. By Theodore A. McGraw, M. D .... 364

ARTICLE VII.—Observations on the Articulation of Artificial Teeth .... 368

ARTICLE VIII.—How to Have Good Teeth .... 372

### EDITORIAL, ETC.

Decision in the Celluloid Case .... 374

Composition in Sozodont, Etc .... 378

### NOTES FROM PRACTICE.

Abscess of Antrum .... 379

A Question of Articulation .... 379

Removing Salivary Calculus from Sub-Lingual Gland .... 380

### MONTHLY SUMMARY.

Status of English Dentistry .... 381

Simple Method of Testing the Purity of Chloroform .... 381

Lionel S. Beale .... 381

External nse of Chloral Hydrate .... 382

American Medical Diplomas Abroad .... 382

The Dangers of Ether .... 383

Making Rubber Stamps .... 384

Chloral in Tetanus .... 384

